# Modeling human disease in rodents by CRISPR/Cas9 genome editing

**DOI:** 10.1007/s00335-017-9703-x

**Published:** 2017-07-04

**Authors:** Marie-Christine Birling, Yann Herault, Guillaume Pavlovic

**Affiliations:** 1 CELPHEDIA, PHENOMIN, Institut Clinique de la Souris (ICS), CNRS, INSERM, University of Strasbourg, 1 rue Laurent Fries, 67404 Illkirch, France; 2 Institut de Génétique et de Biologie Moléculaire et Cellulaire, Université de Strasbourg, 1 rue Laurent Fries, 67404 Illkirch, France; 3 Centre National de la Recherche Scientifique, UMR7104, Illkirch, France; 4 Institut National de la Santé et de la Recherche Médicale, U964, Illkirch, France; 5 Université de Strasbourg, 1 rue Laurent Fries, 67404 Illkirch, France

## Abstract

Modeling human disease has proven to be a challenge for the scientific community. For years, generating an animal model was complicated and restricted to very few species. With the rise of CRISPR/Cas9, it is now possible to generate more or less any animal model. In this review, we will show how this technology is and will change our way to obtain relevant disease animal models and how it should impact human health.

## Introduction

Genome editing and especially the easy and accessible CRISPR/Cas9 technology have open new opportunities in modeling human diseases. Genome-Wide Association Studies (GWAS) with specific point mutations, mutation in coding and non-coding genes, copy number variants (CNVs), and regulatory mutations are now feasible in more or less any genetic background and any species. In this review, we will focus on the latest advancements in the development of disease model in rodents. Because of their phylogenetic relatedness and physiological similarity to humans, their maintenance facility, and easy breeding in the laboratory, mice and rats are the most widely used organisms in research. Genetically engineered rodents allowed major discoveries but sometimes failed to translate to human.

## CRISPR/Cas9 to develop better animal models of human disease

Even if animal models like mouse and rat allowed major breakthrough in biomedical research, a striking issue in modern biology is certainly some failure of mice and other model organism studies to be replicated or translated to humans (Buffenstein et al. 2014; Collins and Tabak 2014; Justice and Dhillon 2016; Pound and Bracken 2014; Young 2013). The predominant utilization of mouse—which represent 61% of the animal used in research in Europe (European Commission 2013)—may be one explanation as mice may respond to experimental interventions in ways that differ strikingly from humans (Perlman 2016). Improper data analysis is also a key factor that limits reproducibility and validity of preclinical mouse research (Kafkafi et al. 2017). The extensive use of few strains like C57BL/6 and 129 substrains (129 mice are a complex collection of various backgrounds (Simpson et al. 1997)) has certainly contributed to this failure too. There is plenty of literature showing that the inbred genetic background has an effect on the phenotype (Bilovocky et al. 2003; No 1997), this clearly demonstrates that a unique mouse inbred strain cannot mimic the outbred diversity of human beings. More recently, Sittig et al. beautifully illustrated that genetic background limits generalizability of genotype–phenotype relationships (Sittig et al. 2016). In the MGI database, C57BL/6 lines (congenic or coisogenic) represent 68% of the >28,000 lines available (MGI extract, February 2017). Mainly due to the fact that only few ES cells lines from specific genetic background (mostly C57BL/6N and 129) are germline competent, most of the phenotyping analyses were done in one of these genetic contexts, mixed backgrounds or 129 models were backcrossed to C57BL/6. Indeed, 129 substrains provide an unfavorable genetic background for some experiments, as most substrains are characterized by poor reproductive performance, neuroanatomical and behavioral abnormalities (Eisener-Dorman et al. 2009).

The use of CRISPR/Cas9 is opening completely new opportunities as it is now possible to generate mutant in almost any genetic background and in various species. In rodents, the only limitation to CRISPR is the knowledge of assisted reproductive techniques (ART) in a given species. The capacity to recover fertilized eggs, to perform the microinjection (in cytoplasm or pronucleus) and to implant them in pseudo-pregnant females is indeed needed to perform CRISPR/Cas9 editing. With a minimal set of ART, It is thus now possible to obtain specific mutation in a scientifically selected genetic background in order to obtain better model. For example, Li et al. achieved high rate Fah gene targeting in *NOD-SCID-IL2RgammaC-null* (NSG) mutant (Li et al. 2014) combining CRISPR and in vitro fertilization. These mice are critical for efficient engraftment of human cells or tissues. Of course, some backgrounds or species are still reluctant to these manipulations. For instance, in Arvicanthis ansorgei, a diurnal rodent widely used for the study of circadian rhythms (Hubbard et al. 2015) it is still not possible to assess when fertilization occurs and when fertilized eggs can be recovered for microinjection (personal communication). Another limitation is, of course, the availability of the genomic sequences as a good CRISPR strategy can only be developed when the whole-genome sequence is known as the specificity of a sgRNA has to be assessed.

Creating a single-nucleotide polymorphism (SNP) animal model of human disease by CRISPR/Cas9 genome editing is now routine in rodent. These models lead to functional insights into the human genetics and allow development of potential new therapies. For example, a human GWAS identified a potential pathological SNP (rs1039084 A > G) in the *STXBP5* gene, regulator of platelet secretion in humans. This mutation was then reproduced by CRISPR in the mouse with the nearly same thrombosis phenotype allowing to confirm the causality of this SNP in human (Zhu et al. 2017). Likewise, whole-genome sequencing was used to perform a GWAS in a population-based biobank from Estonia. A number of potential causal variants and underlying mechanisms were identified. One of them is a regulatory element that is necessary for basophil production, it acts specifically during this process to regulate expression of the transcription factor CEBPA. This enhancer was perturbed by CRISPR/Cas9 in hematopoietic stem and progenitor cells demonstrating that it specifically regulates CEBPA expression during basophil differentiation (Guo et al. 2016).

CRISPR/Cas9 can also specifically reduce the expression of protein in vivo when heterozygous SNPs are involved in dominant inherited conditions. This has been shown in a humanized model of Meesmann’s epithelial corneal dystrophy (MECD), where a mutation within *KRT12* leads to the occurrence of a novel protospacer adjacent motif (PAM). Injection into the corneal stroma of a specific sgRNA (new PAM) with Cas9 resulted in frame-shift deletions within the mutant *KRT12* allele a resulted to a reduced expression of mutant *KRT12* mRNA and protein (Courtney et al. 2015).

With more than 84.7 million different SNP (Huddleston and Eichler 2016) found by sequencing 2500 human genomes, understanding which one are pathologic, neutral, or protective is more than a challenge. We are only at the beginning of understanding SNP function in human and CRISPR/Cas9 genome editing will provide great help to confirm the function of human GWAS-selected SNPs. Indeed, it is now possible to easily introduce specific SNPs in inbred (uniform) and outbreed (heterogeneous) with the help of CRISPR/Cas9.

**Fig. 1:**
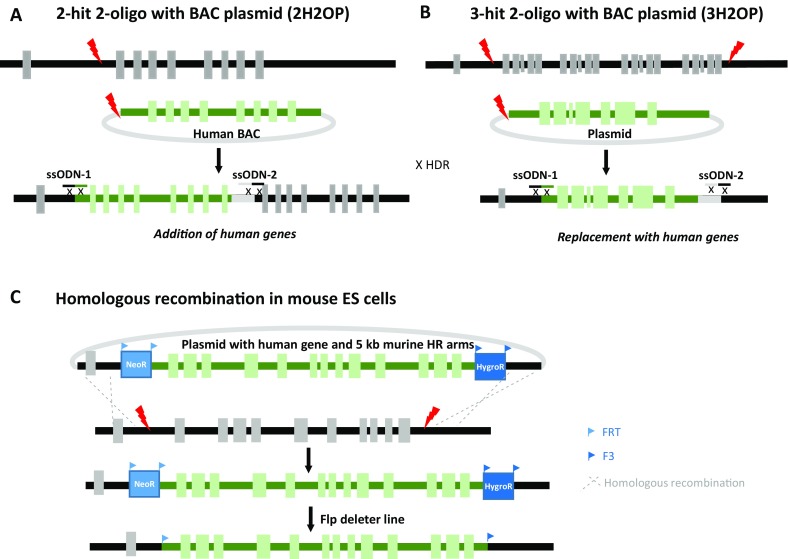
Humanization of whole genomic fragment. **a** Schematic representation of the BAC knock-in by 2-hit 2-oligo. The endogenous gene is knocked out by the insertion of a >100-kb BAC (Knock-In), which contains the human locus. Two sgRNAs targeting the human BAC and the genome to modify are co-injected with 2 ssODNs which have an homology for both the BAC and the genomic DNA (Yoshimi et al. 2016). **b** Schematic representation of the 3-hit 2-oligo method to replace a rat cluster genes (>58 kb), with the orthologous human gene (6.2 kb). Three gRNAs for upstream and downstream of the rat cluster and for the human gene containing plasmid cut the targeting sites, and two ssODNs ligate to each cut end (Yoshimi et al. 2016). **c** Schematic representation of a humanization in mouse ES cells. Two sgRNAs leading to double-strand break 5′ and 3′ of the region to humanize highly increased the number of recombined clones. Well-characterized ES cells clones can then be processed through germline

Humanization of whole genomic fragments is becoming easier. Addition of human sequences as well as replacement of rat sequences by human sequence by straight injection into fertilized eggs was one of the achievements of Yoshimi and collaborators (Yoshimi et al. 2016) (see Fig. 1a, b). A knock-in of a 200-kb human BAC containing the human SIRPA locus, concomitantly knocking out a rat gene was obtained by combining CRISPR/Cas9 (two sgRNAS) with single-stranded oligodeoxynucleotides (Yoshimi et al. 2016). A gene replacement approach using 3 sgRNAs was also achieved. The success rate seems however to be low (1 positive pup for 130 embryos injected, 1 out of 23 offspring) and one of the disadvantage we might see is that the BAC backbone remains present in the rat genome. In our lab, we developed a hybrid approach to humanize a large genomic fragment by using a dual-sgRNAs approach combined to homologous recombination of a targeting construct baring the human sequence with 5-kb mouse homologous arms and two selection markers in ES cells (see Fig. 1c). The murine region is deleted by the two double-strand breaks (DSBs) and some clones undergo homologous recombination repair with the supplied circular vector leading to the humanization of the locus. The frequency of these humanization events is increased by the addition of selection cassettes at both extremities of the human sequences. We have been able to humanize this way a 40-kb region in ES cells (Fig. 1c) and confirm germline transmission for the first model. Without CRISPR, the frequency of these HR events would have been very low (if not null). With CRISPR, 30 (out 186 screened clones; 16.1%) were humanized.

## Understanding the human structural variations that leads to disease

Structural variants (SVs) are large genomic alteration that involves segments of DNA greater than one kilobase (Freeman et al. 2006). Copy number variants (CNVs) are a subfamily of SV that corresponds only to deletion or duplication and do not include inversion or translocation (Freeman et al. 2006). SVs and particularly CNVs are known to be associated with neuropsychiatric diseases (Cook and Scherer 2008; Girirajan et al. 2011). Some CNVs may be larger than 100 kb, although CNVs of this size typically occur at low frequency in the population. Multiple genes and regulatory regions can be affected in CNVs of all sizes (Dasouki et al. 2011; Itsara et al. 2009; Torres et al. 2016). SVs likely play a major role in various diseases not only restricted to neuronal disorders (Conrad et al. 2010; Fanciulli et al. 2007; McCarroll and Altshuler 2007; Wu and Hurst 2016). To date, more than >60,000 SVs were discovered in human (Huddleston and Eichler 2016). Out of more than 60,000 SVs, the ones that are pathological SVs are mostly not known. Likewise, the underlying mechanisms of SVs diseases, even for neuropsychiatric diseases, remain poorly understood. Most of our actual knowledge is based on molecular data from human genome sequencing lacking functional validation.

Chromosome engineering led to a series of mouse models that precisely mimicked the genetic architecture of human patients. In many cases, the approach enabled the etiology of the disease to be linked to individual or small groups of genes. Modeling SV has been achieved so far by using the Cre-lox technology in combination with ES cells or alternative strategies (Adams et al. 2004; Ruf et al. 2011; Zheng et al. 1999) only in mouse. This complex and time-consuming strategy requires the generation of two mouse lines with loxP site followed by 3-step breeding to introduce a Cre driver expressed in the germline (Hérault et al. 1998; Ramírez-Solis et al. 1995; Spitz et al. 2005). Moreover, this approach was not adaptable to rats which are relevant and highly complementary behavior models mainly due to the absence of stable germline-competent ES cells. In mouse, most of the models were analyzed in the C57BL/6 genetic context.

With recent advances in genomic engineering via the use of CRISPR technology, it is now feasible to dissect SV diseases and identify individual genes contributing to their phenotypes, especially for neuropsychiatric disorder SVs. Indeed, with the genomic data connecting SVs with a multitude of human neuropsychiatric disease, our current technical ability to model such chromosomal anomalies in mouse and rat (Birling et al. 2017) and the existence of precise behavioral measures of endophenotypes argue that the time is ripe for systematic dissection of the genetic mechanisms underlying such disease.

### CRISPR/Cas9 and in vitro SVs models

CRISPR/Cas9 genome editing can also be used to achieve interesting in vitro SVs models. One example is the study of recurrent SVs in human-induced pluripotent stem cells (iPSCs). Recurrent SVs arise because of the presence of repetitive sequences within the genome, known as low copy repeats (LCRs). LCRs are stretches of DNA that are typically 10–500 kb (though their size can vary), with greater than 90% sequence identity to another place in the genome (Bailey et al. 2001). The presence of LCRs puts the genome at risk for chromosomal rearrangements that can cause CNVs (Stankiewicz and Lupski 2002). These rearrangements occur because LCRs serve as substrates for non-allelic homologous recombination (NAHR) (Sasaki et al. 2010), which is a recombination between two highly similar DNA sequences that are not alleles (Shaw 2004). NAHR event may result in deletion, duplication, or inversion of large DNA fragment (Turner et al. 2008). Recent in vitro manipulations in human iPSCs reported the use of CRISPR to model the recurrent CNVs 16p11.2 and 15q13.3 (Tai et al. 2016). Reciprocal CNV of a small segment of chromosome 16p11.2 (OMIM 611913) is a common recurrent microdeletion and microduplication syndromes (rMDS) that have been associated with intellectual disability, autism spectrum disorder, schizophrenia, and other neuropsychiatric disorders, as well as anthropometric traits, including obesity (Maillard et al. 2015). 16p11.2 rMDS involves gain or loss of a unique genic segment and one copy equivalent of the segmental duplication. The unique genic segment of the 16p11.2 CNV spans 593 kb (Weiss et al. 2008) containing 47 genes, of which 28 are annotated as protein coding (based on Ensembl GRCh37). It is flanked by parallel and highly homologous (>99% identity) segmental duplications, each spanning 147 kb (Weiss et al. 2008) and containing 6 duplicated genes (Weiss et al. 2008). Tai et al. (2016) obtained rMDS in iPSCs by using two sgRNAs to delete a 575-kb region (one sgRNA targeting both extremities) and an unique sgRNA to target the segmental duplications promoting this way a model of non-allelic homologous recombination (NAHR)-mediated CNV (740 kb) mimicking the consequences of NAHR. This approach will enable modeling of rMDS in multiple tissue types and, with further development and optimization, could provide a tractable route to in vitro correction of these common genomic imbalances.

### CRISPR/Cas9 and in vivo SVs models

In the past 2 years, CRISPR/Cas9 was used to manipulate large genomic region in the mouse genome. The Wu lab (Li et al. 2015) demonstrated that DNA elements of up to 29 kb can be manipulated directly in mice. Zheng and Li’s lab showed that deletion of up to 95 kb can be generated in mice (Wang et al. 2015; Zhang et al. 2015). Similarly, gene clusters of up to 800 kb have been deleted, duplicated, and inversed in mouse ES cell (Kraft et al. 2015). CRISPR/Cas9 was used for the generation of up to 1 Mb structural deletion and inversion around the Tyrosinase locus in mouse zygotes but duplications appeared less frequently and did not pass the germline (Boroviak et al. 2016).

**Fig. 2:**
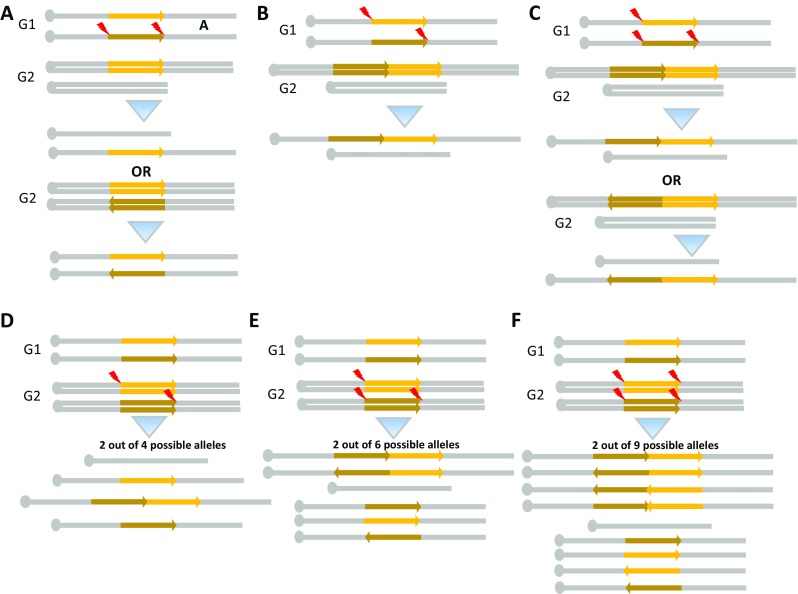
CRISpr-MEdiated REarrangement mechanisms (CRISMERE). **a–c** Standard chromosomic recombination when Cas9 edits the genome in G1. After mitosis, two alleles distinct from the initial WT allele will be obtained. **a** Intra-chromosomal recombination between two DSBs on a single chromosome. **b** Trans-allelic recombination between two DSBs each on one of the two chromosome. **c** Trans-allelic recombination between three (or four DSBs) on the two chromosomes ending with head to head, tail to tail duplication. **d–f** Standard chromosomic recombination when Cas9 edits the genome in G2. **d** Schematic of the event that should take place in the eggs where Cas9 edits the genome in *cis* configuration in G2 leading to monosomic and trisomic daughter cells after mitosis. Trans-allelic recombination between two DSBs on the two chromatids in G2. **e** Trans-allelic recombination between three DSBs when two breaks are located on only one chromatid and the third break is located on another chromatid. **f** Trans-allelic recombination between four DSBs when two breaks are located on one chromatid and the two other are located on another chromatid. As much as nine possible alleles can be obtained

Very recently, we have demonstrated that CRISPR/Cas9 genome editing allows to generate easily and quickly any type of SV mutations in mouse and also in rat (Birling et al. 2017). The variety of SVs which can be obtained is depicted in Fig. 2. Our data suggest that the timing of CRISPR/Cas9-mediated DSB during the growth phase G1 or G2 of the cell division cycle is certainly changing the outcomes. DSB occurring during the G2 phase will lead to an impressive potential variety of alleles (see Fig. 2). We have obtained up to six different alleles for one single founder. Indeed, animal models generated via zygotic injection of CRISPR reagents are often genetic mosaics (Yen et al. 2014), com pers & data not shown). These animals are thus composed of cells (somatic but also germinal) that may carry different mutations in the target allele. Of course, it is impossible to define if these arose from the mitosis of one cell or are the results of the action of CRISPRs at the 2-cell stage. We (Birling et al. 2017) have been able to generate Down Syndrome models by duplicating the syntenic region in both rat and mouse. The largest region we managed to duplicate is 24 Mb in the rat corresponding to the main human chromosome 21 syntenic region located on chromosome 11 in the rat. Established mouse and rat lines have been obtained in which regions as large as 4.9 Mb are duplicated (resulting of three copies of the region) or deleted (resulting of one copy of the region). Inversions also occurred at a good frequency. The only limit for obtaining a specific SV is the viability of the mutant cells and of the organism. For example, the deletion of the 24-Mb Lipi-Zfp295 genomic region does not seem viable in mouse. We were able to observe this deletion in newborn rats but they died quickly. Of course, chromosomal translocations can be made by designing CRISPRs to appropriate breakpoints on distinct chromosomes. Finally, we have optimized our CRISPR protocol and shown that four CRISPRs (two pairs at the desired breakpoints) are even more efficient to generate SVs. With our protocol, the efficiency for generating SV mutations seems as good as what we observed for the generation of a critical exon knock-out by CRISPR injection in eggs (on eight projects, we obtained 5–80% of the founders with the awaited deletion, inversion or duplication)(Birling et al. 2017). The use of ES cells can be easily avoided for the generation of SVs.

### CRISPR/Cas9 and SVs cancer models

Somatically acquired SVs can induce alterations in genes that directly contribute to cellular transformation (Feuk et al. 2006). Again CRISPR/Cas9 genome editing changes the game to study such diseases. An in vivo model of somatically acquired SV human lung cancer has been generated by Maddalo et al. by viral delivery of CRISPR/Cas9 to somatic cells of adult mice to achieve an 11-Mb inversion. Two sgRNAs were used to generate the 11-Mb inversion on the short arm of chromosome 2: inv(2)(p21p23) leading to the expression of the echinoderm microtubule-associated protein like 4-anaplastic lymphoma kinase (*EML4–ALK*) oncogene in a small subset of human cells. The resulting cells invariably harbor the *Eml4–Alk* inversion, express the Eml4–Alk fusion gene, display histopathological and molecular features typical of *ALK1* human non-small cell lung cancers (NSCLCs), and respond to treatment with ALK inhibitors (Maddalo et al. 2014). In the case of this experiment, low efficiency of viral delivery and CRISPR-mediated inversion events are an advantage allowing that only a subset of somatic cells are modified and so recapitulating the stochastic nature of tumor formation in humans.

## CRISPR/Cas9 therapeutic applications

For the past few years, CRISPR/Cas9 gene editing technology has become essential for the generation of animal model of human diseases. CRISPR genome editing is now also under evaluation for therapeutic applications like cancer immunotherapy, tissue regeneration, gene therapy, HIV and viral disease, and obesity and metabolism.

The most striking example is the exon skipping approach developed on Duchenne Muscular Dystrophy (DMD) mice. DMD is one of the most prevalent fatal genetic diseases, with no successful, long-term treatments currently available. It is caused by any of a large spectrum of mutations in the Dystrophin gene that lead to loss of functional protein making it a prime candidate for editing by CRISPR/Cas9. Although many genetic therapeutic approaches for DMD have been attempted over the years, success has been very limited, in part by the large size of Dystrophin and the difficulty of achieving long-term rescue. Since 62% of DMD patients have mutations in exons 45–55 of Dystrophin, targeting this non-essential region to restore the open reading frame (ORF) by exon skipping has been a compelling strategy (Ousterout et al. 2015). Following proof-of-principle studies, several groups recently reported Cas9-mediated gene editing in vivo using the mdx mouse model of DMD, which contains a natural mutation in exon 23 of Dystrophin. Using adeno-associated virus (AAV) delivery, all three groups targeted Cas9 to the exon 23 splice junctions in Dystrophin, taking advantage of repair by non-homologous end joining (NHEJ) to delete the mutated exon and restore the ORF. The clinical impact of this technology is that genome editing can permanently correct disease-causing mutations and circumvent the hurdles of traditional gene- and cell-based therapies (Long et al. 2016; Nelson et al. 2016; Tabebordbar et al. 2016a). In all three reports, Dystrophin expression was restored to therapeutic levels in the affected muscles and the dystrophic phenotype was improved. Following muscle-tropic AAV9 delivery of Cas9 components in the mdx mouse, a small percentage of muscle satellite cells displayed evidence of gene editing (Tabebordbar et al. 2016a), and numbers of dystrophin-positive myofibers increased over time (Long et al. 2016), consistent with editing of satellite cells. These mouse models are good proof of concept for potential gene therapy in human but of course, the development of Cas9-based technologies into a therapeutic approach for DMD will require advances on several fronts, including tissue delivery, increased efficiency of genome editing/modification and technical improvements in the stability, specificity, and delivery of Cas9 components.

## Using CRISPR/Cas9 to generate thousands mouse models improving knowledge on protein-coding genes

The International Mouse Phenotyping Consortium (IMPC) (http://www.mousephenotype.org/) builds on the efforts of research institutions worldwide to produce knock-outs of protein-coding genes and carry out high-throughput phenotyping of these mutants. The ultimate goal is to determine the function of every gene in the mouse genome (Brown and Moore 2012). These mice are preserved in repositories and made available to the scientific community representing a valuable resource for basic scientific research as well as generating new models for human diseases. Nearly 6000 mouse lines have already been generated in pure C57BL/6 N background through homologous recombination in ES cells and most of them have a ‘knockout first, conditional ready’ allele. This clever allele allows the generation of a lacZ-tagged null allele or a conditional allele, respectively (Bradley et al. 2012), by a simple breeding with Cre or Flp delete lines. The phenotyping information for more than 3000 protein-coding genes has been made freely available online to the scientific community. This initial work has confirmed that about one-third of these genes are essential for life, and has provided phenotyping information for many genes with unknown function, helping to piece together gaps in our understanding of the genome (Dickinson et al. 2016; de Angelis et al. 2015). The database allows scientists to research a gene of interest and provides crucial insight into the underlying causes for rare and common diseases. To date, more than 4000 human diseases are associated with IMPC mouse models (IMPC website, March 2017). Each mutant mouse line are tested through a broad-based primary phenotyping pipeline in all the major adult organ systems and most areas of major human diseases. Phenotyping tests are standardized and cross validated between centers of the consortium (Simon et al. 2013) for decreasing the percentage of non-replicable discoveries. It is important to point out here that heterozygous KO mouse lines are phenotyped when homozygous KO animals are lethal or subviable. The IMPC web site is really user friendly and allows to search by gene, phenotype, embryonic phenotyping, gene interaction but also by human diseases which renders this site particularly interesting for human geneticists (http://www.mousephenotype.org/). A search is possible by OMIM reference. The known genes implicated in a specific disease are registered and all potential mouse models with phenotypic similarities are registered by similarity scores (Rosen et al. 2015).

Since 2014, limited by the availability and quality of targeted ES cells and also because of its ease of use and cost, the IMPC members have decided to switch to CRISPR to generate knock-out (KO) alleles. The choice was to obtain straight KO of all genes as CRISPR has been shown to be very efficient whatever the genomic complexity and the generation of such line will be at lower cost. More than a 1000 lines have already been generated by this approach. Most of the KO alleles are the deletion of one (or more) critical exon(s). The only limit is the founder’s viability if sgRNAs are too efficient (high number of homozygous KO cells or haplo-insufficient genes). Indeed, we have shown that approximately 30% of full-gene KOs die during embryogenesis or at early post-natal days (Dickinson et al. 2016; de Angelis et al. 2015). A paper describing in more detail this work is currently under redaction.

## Non-coding genetic and regulatory elements

Despite the overwhelming number of human non-coding RNAs reported so far, little is known about their physiological functions for the majority of them (Table 1). On their website, the ENCODE project, an international consortium that aims to build a comprehensive parts list of functional elements in the mammalian genomes, estimates that the human and mouse genome contains 23,025 and 16,592 non-coding genes, respectively. In the Mouse Genome Informatics resource (MGI, http://www.informatics.jax.org/), the most exhaustive database for the laboratory mouse, only 161 (1.0%) non-coding genes knock-out mouse models have been generated and 146 (0.9%) of them have a phenotype described. No more data exist on the other regulatory elements. For example, it is estimated that the human genome contains >500,000 putative enhancers, a staggering number that poses a major challenge for the identification of functional regulatory elements (Korkmaz et al. 2016).

**Table 1:** Number of predicted genes in human and mouse genomes and correlation with functional data extracted from MGI

	In human^a^	In mouse^b^	With a phenotype described in mouse (in % OF potential genes)^c^	With more than one phenotype described in MGI (in % of potential genes)^c^
Protein-coding genes	19 950	21 973	9 745 (44.3%)	5 387 (24.5%)
Non-coding genes	23 025	16 592	146 (0.9%)	78 (0.5%)
Pseudogenes	14 650	10 524	8 (0.1%)	4 (0.0%)

^a^Statistics from GENCODE Human version 25 release (March 2016)

^b^Statistics from GENCODE Mouse version 14 release (August 2016)

^c^Analysis done using the phenotyping annotations available in MGI on February 2017

Lack of knowledge is obvious and very few examples of CRISPR/Cas9 gene editing of non-coding genes or other regulatory elements can be found. The long non-coding RNA *AK023948* is an example of how CRISPR/Cas9 can be used to decipher non-coding gene function (Koirala et al. 2017). *AK023948* knock-out in human MCF-7 cells suppresses the AKT activity, a critical pathway involved in cell survival, growth, proliferation, angiogenesis, metabolism, and cell migration. The potential impact of *AK023948* in the promotion of tumorigenesis is clearly highlighted (Koirala et al. 2017).

## CRISPR/Cas9, polygenic disorders, and personalized medicine

Genetic disorders that are caused by the combined action of more than one gene are another layer of the genome complexity. Common human diseases or traits—such as size, diabetes, heart disease, schizophrenia, and autism—are typically polygenic (Gandal et al. 2016; Marouli et al. 2017; Roberts et al. 2013; Sharma and Vella 2017). A variety of genetic resource are available in mouse such as recombinant inbred lines (Carneiro et al. 2009; Williams et al. 2001), consomics (Gregorová et al. 2008), heterogeneous stocks (Valdar et al. 2006), and the Collaborative Cross (Churchill et al. 2004). These resources are powerful tools to study polygenic diseases but have been underutilized because genetic modifications in these strains were very complex. Whereas obtaining germline transmission from a fully validated ES cells can sometimes be difficult, strictly due to the intrinsic germline competency of the ES cells (and not to the engineered allele), this is not anymore an issue when CRISPR is injected straight into the eggs. Fertilized eggs of almost any genetic background can be recovered, microinjected (alternatively an electroporation) with CRISPRs and implanted. Specific mutations can be introduced in any genetic background. For example, in NOD mouse, a model of spontaneous type 1 diabetes (T1D), a R619W mutation in the protein tyrosine phosphatase non-receptor type 22 (*Ptpn22*) gene was introduced by CRISPR/Cas9 and homology-directed repair (Lin et al. 2016). This mutation corresponds to the human allelic variant of *PTPN22* (R620W), an allele strongly associated with type 1 diabetes (T1D) which increases the risk of T1D by two- to fourfold. The resulting *Ptpn22* (R619W) mice showed increased insulin autoantibodies, earlier onset and higher penetrance of T1D. This is the first report demonstrating enhanced T1D in a mouse modeling human *PTPN22* (R620W), it shows the utility of CRISPR/Cas9 for direct genetic alternation of NO D mice. It is now possible to study polygenic disorders in various genetic contexts and this gives new opportunities for personalized medicine in human. By this ability to study a gene in various genetic contexts, CRISPR may also be highly valuable to study incomplete penetrance and variable expressivity as these traits probably result from a combination of genetic and environmental factors.

## Will CRISPR genome editing allow to decrypt the human genome?

Sixteen years have passed since the initial sequencing and analysis of the human genome was published by International Human Genome Sequencing Consortium (Lander et al. 2001; Venter 2001). It immediately brings high expectations for improvements in the treatment of common disorders and strategies for the prevention of disease. However, our genome (and the genome of the other mammals) remains poorly decrypted and the effort of high-throughput programs like the IMPC to discover and ascribe biological function to each gene is more than ever required.

First, the function of half of the protein-coding genes is not known (Table 1). Pleiotropy is poorly assessed for most of the coding genes but however seems to be the rule. The analysis of 449 knock-out mouse mutant models by the EUMODIC consortium show that 65% of the corresponding genes are pleiotropic (de Angelis et al. 2015). Likewise, very little is known about the number and function of non-coding genes (Table 1). For example, the GENCODE project estimates that the human genome contains many thousands of long non-coding RNAs (Derrien et al. 2012). Again, the function of the vast majority of these potential genes remains to be decrypted (Table 1). Finally, the “remaining” part of the mammalian genome (pseudogenes, repeated sequences, desert islands …) may not be accurately considered as junk DNA. For example, there is emerging evidence that many of the pseudogenes could be biologically active (Frankish and Harrow 2014). In a few cases what was named a pseudogene was indeed having a concrete function (Barau et al. 2016). Similarly, the ENCODE project assigned potential biochemical functions for 80% of the human genome (Dunham et al. 2012) raising two questions:

1. Which of these active genomic elements code for a real biological function or is it just noise?
2. Do some of these sequences have evolutionary functions?

Today, most of our knowledge on the mammalian genomes is based on bioinformatics analyses of large set of molecular data. In a world where the sequencing of genomes is becoming cheap and fast, how can we decrypt the function of the human genome and its genomic variations? We believe that CRISPR/Cas genome editing is the Swiss knife for functional studies. By using more wisely animal models, CRISPR/Cas9 will be a tool to link human genome to diseases and phenotypes. Now, the main limit is not to obtain an animal model: the choice of the relevant background and species to mimic a human pathology is the challenge.

Finally, as the ultimate goal of most animal models is humanization and as the CRISPR technology gives now the tools to perform more or less any genomic modification, one might step back and ask which humanization strategy is the best. For example, introduction of an orthologous (causal) mutation can lead to a ‘better’ model than a whole gene humanization. Indeed, genes have evolved from a common ancestral gene by speciation and we know that orthologs generally retain the same function in the course of evolution. The introduction of a human disease causative mutation in an orthologous rodent gene very often leads to the similar phenotype. We cannot anticipate how a human gene will behave in another species, regulatory sequences can be located in introns and 5′ and 3′ sequences. Do we have to keep the human regulatory sequences or is it better to keep to host species regulatory elements. It is really important to keep the human introns or is it better to use a cDNA for a faithful expression of a gene? How will a humanized protein interact with its rodent counterpart(s)? How will it interact in complexes or pathways? A case by case study is certainly the answer. Now that the genetic tools are available, any relevant animal model of human disease seems possible.
